# Strontium-doped hydroxyapatite microspheres loaded with iloprost promote dentin–pulp complex regeneration

**DOI:** 10.3389/fbioe.2025.1726285

**Published:** 2026-01-14

**Authors:** Jilong Chen, Jingyi Di, Qiming Yu, Hui Xiao, Ting Wang, Li He

**Affiliations:** Department of Stomatology, Renmin Hospital, Hubei University of Medicine, Shiyan, Hubei, China

**Keywords:** angiogenesis, human dental pulp stem cells (hDPSCs), hydroxyapatite hollow microspheres, iloprost (PGI_2_ analog), reparative dentinogenesis, strontium doping, vital pulp therapy (VPT)

## Abstract

**Background:**

Vital pulp therapy (VPT) aims to preserve pulp vitality and tooth function. However, materials like calcium hydroxide and mineral trioxide aggregate have limitations in bioactivity, underscoring the need for improved biomaterials. Strontium-doped hydroxyapatite (Sr-HA) and pro-angiogenic agents have emerged as promising strategies to enhance dentin–pulp complex regeneration.

**Methods:**

Hollow hydroxyapatite microspheres with 5%, 10%, and 15% Sr substitution were synthesized, and the optimal concentration was identified through Sr^2+^ release profiling and CCK-8-based cytocompatibility screening. Iloprost was subsequently loaded onto the selected 5% Sr-HA to obtain Ilo@Sr-HA. Human dental pulp stem cells (hDPSCs) were isolated from healthy extracted premolars using the tissue-explant method and identified by flow cytometry and multilineage differentiation assays. The identified cells were used to assess viability, ALP activity, mineralized nodule formation, and odontogenic gene expression. A bilateral rat pulp-exposure model (N = 40; n = 10/group: Blank, Dycal, Sr-HA, Ilo@Sr-HA) was established. Reparative outcomes were quantified using micro-CT and histological scoring at days 7 and 28.

**Results:**

Preliminary screening identified 5% Sr-HA as optimal, with the best ion release and cytocompatibility. Ilo@Sr-HA showed a biphasic release and no cytotoxicity toward hDPSCs. *In vitro*, Ilo@Sr-HA enhanced hDPSCs proliferation and ALP activity compared with HA and Sr-HA. Mineralized nodule formation increased, with significant DMP1 and DSPP upregulation (P < 0.05). *In vivo*, Ilo@Sr-HA enhanced reparative dentin formation, with DV/TV reaching 38.91% at 4 weeks vs. 26.53% for Dycal (P < 0.01). Histology confirmed continuous dentin bridges in the Ilo@Sr-HA group, contrasting with incomplete structures in Dycal and Sr-HA. Lower inflammation and better pulp preservation were also observed.

**Conclusion:**

Ilo@Sr-HA combines Sr^2+^ ionic cues with iloprost’s pharmacological effects to form a bioactive microenvironment that supports pulp repair and reparative dentinogenesis. Ilo@Sr-HA is a promising material for VPT and dentin–pulp regeneration.

## 1 Introduction

Vital pulp therapy (VPT) aims to preserve the vitality of the dental pulp in affected teeth, allowing it to maintain its physiological functions, thereby retaining the natural tooth and improving both treatment prognosis and patient quality of life (Colloc and Tomson, 2025; Zhang L. et al., 2025). Novel pulp-capping agents represent a major research focus in VPT) (Hong et al., 2020; Li et al., 2025; Rajasekar et al., 2025). Traditional materials, such as calcium hydroxide and mineral trioxide aggregate (MTA), are widely used; however, their inherent limitations—including insufficient sealing ability, high solubility, limited bioactivity, and the risk of tooth discoloration—pose significant challenges to the long-term success of VPT (Singer et al., 2023; Al-Saudi, 2024; Priyadharshini et al., 2024; Abuhashema et al., 2025). To address these drawbacks, newer bioceramic materials such as Biodentine®, BioAggregate, TheraCal LC and Super MTA Paste have been developed to improve clinical outcomes (Islam et al., 2023). For example, Biodentine exhibits excellent sealing properties and stimulates dentin bridge formation, while Bio Aggregate, being an aluminum-free material, shows high biocompatibility and promotes favorable tissue healing (Minic et al., 2021; Gangishetti et al., 2024; Lu L, 2025; Silva et al., 2025). However, more regenerative and biologically responsive materials are still required for pulp–dentin repair.

Hydroxyapatite (HA), the major inorganic constituent of dentin and bone, is widely recognized for its excellent biocompatibility and osteoconductivity (Hsiung et al., 2025). Accordingly, synthetic HA has been extensively applied as a scaffold and as a carrier for therapeutic molecules in regenerative medicine (Munir et al., 2021; Amiryaghoubi and Jahanban Esfahlan, 2024; Hatami kaleshtari et al., 2025). However, in the context of vital pulp therapy, an ideal biomaterial must not only support cell adhesion and proliferation but also actively induce odontogenic differentiation, mineralization, and reparative dentin bridge formation. To address these clinical requirements, various ion-substitution strategies have been developed to enhance the bioactivity of HA (Kurzyk et al., 2023; Paramasivan et al., 2023; Salem et al., 2025). Among them, strontium-doped hydroxyapatite (Sr-HA) has attracted considerable interest due to its multi-functional biological effects, including promoting osteoblast differentiation, inhibiting bone resorption, and providing antibacterial as well as anti-inflammatory benefits (Tsai et al., 2018; Sheng et al., 2023; Radulescu et al., 2025). Sr-based biomaterials have been shown to elevate alkaline phosphatase (ALP) activity, enhance extracellular matrix mineralization, and upregulate odontogenic/osteogenic gene expression in stem cells (Aimaiti et al., 2017; Liu et al., 2023a; Zhang Y. et al., 2025), partly through activation of regeneration-associated pathways such as Wnt/β-catenin and MAPK (You et al., 2022; Wang et al., 2025). These advantages highlight Sr-HA as a promising candidate for pulp-capping applications. Nevertheless, while HA provides a favorable biological foundation, further optimization through compositional and structural modification may be required to maximize its regenerative performance.

In addition to compositional modifications, tailoring the structural features of HA represents another effective strategy for enhancing its biological performance (Jiang et al., 2022; Diez-Escudero et al., 2023). Particularly, structural designs that enable controlled drug delivery have received increasing attention (Lee et al., 2021; Liu et al., 2021). Among these, hollow hydroxyapatite microsphere serve as efficient platforms for drug loading and sustained release, thereby rendering them promising candidates for therapeutic delivery systems (Zeng et al., 2023). Notably, various bioactive molecules—including dexamethasone, statins, and antibiotics—have been successfully incorporated into hollow hydroxyapatite microsphere, where they effectively suppress inflammation and promote tissue regeneration (Xiao et al., 2016; Wang et al., 2017; Liu et al., 2023b). Iloprost, a stable analogue of prostacyclin (PGI_2_), has been clinically employed in the treatment of pulmonary arterial hypertension and peripheral vascular diseases due to its vasodilatory, microcirculation-improving, and antiplatelet effects (Zhou et al., 2024). It also exerts anti-inflammatory and barrier-protective activities (Filippini et al., 2021). Recent preclinical studies have demonstrated that iloprost enhances angiogenesis, increases pulpal blood flow, and supports cell migration and differentiation in pulp and pulp cell models, particularly in combination with scaffolds or MTA, highlighting its potential as a bioactive agent in pulp regeneration (Seang et al., 2018; Almeshari et al., 2021; Almeshari et al., 2022). However, the available evidence is largely derived from *in vitro* and small animal studies, and its clinical relevance remains to be established. To the best of our knowledge, the use of combining iloprost with Sr-doped hollow hydroxyapatite microspheres (Ilo@Sr-HA) for pulp-capping applications has not been previously reported.

Based on these considerations, we hypothesize that the synergistic effects of Sr doping and iloprost delivery may generate a bioactive microenvironment conducive to pulp repair and regeneration. Therefore, the present study was designed to: (1) synthesize and characterize Ilo@Sr-HA; (2) evaluate their effects on the proliferation, odontogenic differentiation, and mineralization of human dental pulp stem cells (hDPSCs) *in vitro*; and (3) investigate their efficacy in promoting reparative dentin formation in a rat pulp exposure model.

To test these hypotheses, we synthesized, characterized, and evaluated the biological performance of ilo@Sr-HA, as described in the Materials and Methods.

## 2 Materials and methods

This study involved both *in vitro* and *in vivo* experiments conducted at the Department of Stomatology, Renmin Hospital, Hubei University of Medicine, from July 2024 to July 2025.

### 2.1 Preparation of Sr-HA

Hydroxyapatite hollow microspheres with varying strontium doping ratios were synthesized via a one-step hydrothermal method. Stoichiometric amounts of calcium nitrate tetrahydrate (Ca(NO_3_)_2_·4H_2_O, Macklin, China) and strontium nitrate (Sr(NO_3_)_2_, Macklin, China) were dissolved in deionized water to prepare the cationic solution, in which the molar fraction of Sr^2+^ relative to the total amount of (Ca^2+^ + Sr^2+^) was adjusted to 0% (pure HA), 5%, 10%, and 15%. These doping ratios were selected based on preliminary experiments, and are supported by previous reports in bone tissue engineering, where comparable levels of Sr incorporation have been shown to enhance osteogenic activity while maintaining HA phase stability (Hu et al., 2020). Ammonium dihydrogen phosphate ((NH_4_)_2_HPO_4_, Macklin, China) was dissolved in deionized water to obtain the anionic solution.

Under vigorous stirring, the anionic solution was added dropwise into the cationic solution until the final molar ratio of (Ca + Sr)/P reached 1.67. The pH of the mixed solution was then adjusted precisely to 10.0 using ammonia solution. The resulting suspension was transferred into a stainless-steel autoclave lined with polytetrafluoroethylene and maintained at 180 °C for 24 h. After the hydrothermal reaction, the autoclave was cooled naturally to room temperature.

The precipitates were collected by centrifugation and washed several times with alternating deionized water and anhydrous ethanol. Finally, the obtained powders were dried in an oven at 60 °C for 12 h, yielding a series of Sr-HA with different strontium doping ratios (5%, 10%, and 15%).

### 2.2 Materials characterization

The surface morphology and internal hollow structures of the microspheres with different strontium doping ratios were examined using a scanning electron microscope (SEM; Zeiss Sigma 300, Germany). The elemental composition, as well as the actual content and spatial distribution of strontium within the microspheres, was determined by energy-dispersive X-ray spectroscopy (EDS; equipped on Zeiss Sigma 300, Germany). The carbon signal detected by EDS was excluded from the quantification as it primarily originates from the conductive carbon tape used during SEM sample preparation.

The crystal phase structures of the samples were analyzed by X-ray diffraction (XRD; Bruker D8 Advance, Germany) using Cu Kα radiation. Diffraction patterns were recorded in the 2θ range of 20°–60° at a scanning speed of 2°/min. In addition, Fourier transform infrared spectroscopy (FTIR; Bruker Tensor II, Germany) was employed to characterize the functional groups and chemical bonds present on the surface and within the samples.

### 2.3 Characterization and identification of hDPSCs

Human dental pulp cells were obtained from healthy permanent premolars extracted during orthodontic treatment at the Department of Stomatology, Renmin Hospital, Hubei University of Medicine (donor age: 16–20 years). All donors were systemically healthy and free of underlying diseases. The study protocol was approved by the Ethics Committee of Renmin Hospital, Hubei University of Medicine (Approval No.: SYSRMYY-2025-039). Written informed consent was obtained from all volunteers and tooth donors, in accordance with the guiding principles of the Bioethics Law of China.

Human dental pulp stem cells (hDPSCs) were isolated by enzymatic digestion and cultured in DMEM/F12 medium (Gibco, United States) supplemented with 20% fetal bovine serum (FBS; Gibco, United States) and 1% antibiotics (penicillin G, 100 U/mL; streptomycin, 100 μg/mL; Hyclone, United States). When cells reached 70%–80% confluence at passages 2–4 (approximately 15–20 days of culture), they were subcultured, and the FBS concentration was reduced to 10%. Subsequent experiments were performed using hDPSCs from passages 2–4. For phenotypic identification, passage 3 cells were assessed by flow cytometry analysis of surface markers, followed by osteogenic and adipogenic differentiation assays.

### 2.4 Screening the optimal Sr doping ratio in Sr-HA

Freeze-dried Sr-HA powders with 5%, 10%, and 15% strontium doping ratios (20 mg each) were weighed into separate 15-mL centrifuge tubes. Each sample was dispersed in 10 mL of deionized water and placed in a constant-temperature shaker (37 °C, 60 rpm). At days 1, 3, 7, 14, 21, and 28, the tubes were retrieved, and 5 mL of supernatant was collected. An equal volume (5 mL) of deionized water was then replenished into the remaining precipitate. The Sr^2+^ concentration in the supernatants was determined using inductively coupled plasma mass spectrometry (ICP-MS).

To assess the effects of different Sr doping ratios on hDPSCs, cells were seeded at a density of 2 × 10^3^ cells per well in 96-well plates. After 24 h of initial culture, the cells were co-cultured with Sr-HA at doping ratios of 0%, 5%, 10%, and 15%, at a final concentration of 2 mg/mL, and to mimic *in vivo* metabolic dynamics, half of the culture medium was replaced every 2 days (Cheng et al., 2023). At days 1, 3, and 5, CCK-8 solution was added, and the cells were incubated in the dark for 1 h. Absorbance was then measured at 450 nm using a microplate reader to evaluate cell proliferation. Based on these results, 5% Sr-HA was selected for subsequent experiments.

### 2.5 Drug loading and release profiles

Drug loading was carried out using a vacuum impregnation method (Rahmani-Moghadam et al., 2021). Briefly, 100 mg of Sr-HA powder was dispersed in 5 mL of phosphate-buffered saline (PBS; Solarbio, China) containing 1 mg of iloprost (Solarbio, China). The suspension was placed in a vacuum drying oven, evacuated to −0.1 MPa for 30 min to remove air from the microspheres, and then maintained at atmospheric pressure for 12 h. The mixture was centrifuged at 8,000 rpm for 5 min, and the supernatant was discarded. Surface-adsorbed drug was removed by gently washing once with deionized water. Finally, the drug-loaded microspheres were freeze-dried for 48 h to obtain ilo@Sr-HA powder, which was stored at 4 °C in the dark until further use.

For the release study, 20 mg of ilo@Sr-HA powder was placed into a centrifuge tube and incubated in 10 mL of PBS (pH 7.4). The tube was maintained in a constant-temperature shaking incubator at 37 °C and 100 rpm. At predetermined time points (1, 2, 3, 5, 7, 14, and 21 days), 1 mL of release medium was collected and replaced with an equal volume of fresh PBS. The absorbance of the supernatant at 299 nm was measured using a UV–Vis spectrophotometer (TU-1901, Beijing Purkinje, China). Iloprost concentrations were calculated from a standard curve established in PBS, and the cumulative drug release profile was plotted.

### 2.6 *In vitro* evaluation of ilo-Sr-HHAM biocompatibility

The biocompatibility of different material groups with hDPSCs was evaluated through live/dead cell staining and CCK-8 proliferation assays. Five experimental groups were included: blank control (Control), pure HHAM, 5% Sr-HA, iloprost, and ilo@Sr-HA.

#### 2.6.1 Live/dead cell staining

For qualitative assessment of cell viability, hDPSCs were directly co-cultured with each group of materials for 5 days. Cells were stained using the Calcein-AM/PI Live/Dead Cell Double Staining Kit (Solarbio, China). Calcein-AM labels viable cells with green fluorescence, whereas propidium iodide (PI) stains the nuclei of dead cells with red fluorescence. Fluorescence images were obtained using a fluorescence microscope (Leica DM6B, Germany).

#### 2.6.2 CCK-8 proliferation assay

For quantitative evaluation of proliferation, hDPSCs were seeded at a density of 2 × 10^3^ cells per well in 96-well plates and directly co-cultured with the different material groups for 1, 3, and 5 days. At each time point, CCK-8 solution (Beyotime, China) was added, followed by incubation in the dark for 1 h. The absorbance at 450 nm was measured using a microplate reader to determine the proliferation rate in each group.

### 2.7 Validation of Ilo@Sr-HA on the odontogenic and osteogenic potential of hDPSCs

The experimental design was consistent with Section 1.7.

#### 2.7.1 Alkaline phosphatase (ALP) activity assay

After 14 days of direct co-culture, ALP staining was performed using the BCIP/NBT Alkaline Phosphatase Assay Kit (Beyotime, China) according to the manufacturer’s instructions to visualize intracellular ALP expression. In parallel, quantitative assessment was conducted using the Alkaline Phosphatase Activity Assay Kit (Beyotime, China). Briefly, cell lysates were collected and incubated with the substrates provided in the kit under specified conditions, and absorbance at 405 nm was measured to calculate ALP activity.

#### 2.7.2 Mineralized nodule staining (alizarin red S)

After 28 days of co-culture, extracellular matrix mineralization was assessed by Alizarin Red S (ARS) staining. Cells were gently washed 2–3 times with PBS, fixed with 4% paraformaldehyde (Solarbio, China) for 15 min, and rinsed again with PBS. They were then incubated with 1% ARS solution (pH 4.2, Beyotime, China) for 30 min at room temperature. Excess dye was removed by thorough washing with deionized water, and mineralized nodules were observed under a microscope. For quantitative analysis, bound ARS was solubilized in 10% cetylpyridinium chloride solution (Beyotime, China). After shaking at room temperature for 30 min, the supernatant was collected, and absorbance at 562 nm was recorded.

#### 2.7.3 Odontogenic gene expression analysis (qRT-PCR)

After 5 days of co-culture, total RNA was extracted and reverse-transcribed into cDNA. Quantitative real-time PCR (qRT-PCR) was performed using a Bio-Rad CFX96 system (Bio-Rad, United States) with SYBR Green Master Mix to assess the expression of odontogenic markers including DMP1, DSPP, and ALP, with GAPDH as the internal control. Relative expression levels were analyzed using the 2^−^ΔΔCt method. The sequences of primers used for qRT-PCR are provided in Supplementary Table S1.

### 2.8 Animal models and surgical procedures

All animal experiments were approved by the Animal Ethics Committee of Hubei University of Medicine (Approval No. HBMU-2025-36) and conducted in accordance with the ARRIVE guidelines and the 2021 PRIASE (Preferred Reporting Items for Animal Studies in Endodontology) recommendations. A total of 40 healthy male Sprague–Dawley (SD) rats (8 weeks old, 250–300 g) were randomly assigned into four groups (n = 10 per group) using a computer-generated random number table (Excel 2016, Microsoft Corp.).

1. Blank Control Group: No material was applied.
2. Standard Control Group: Dycal powder was applied to the exposed pulp and immediately covered with glass ionomer cement.
3. Sr-HA Group: Sr-HA powder was applied to the exposed pulp.
4. Ilo@Sr-HA Group: Ilo@Sr-HA powder was applied to the exposed pulp.

Each rat received pulp exposure on both maxillary first molars, resulting in 20 treated teeth per group. General anesthesia was induced by intraperitoneal injection of 3% sodium pentobarbital (40 mg/kg,Solarbio, China), and postoperative analgesia was provided with subcutaneous meloxicam (1 mg/kg; Solarbio, China) once daily for 2 days. The pulp chambers of the maxillary first molars were exposed using a dental turbine handpiece with a #1/4 round bur under continuous water cooling, creating an exposure site of approximately 0.5 mm in diameter. The cavities were irrigated with sterile saline, and hemostasis was achieved using sterile cotton pellets. Finally, in all groups, the cavities were coronally sealed with FXII glass ionomer cement.

### 2.9 Imaging and histological evaluation

#### 2.9.1 Sample collection and micro-CT analysis

At 7 and 28 days post-surgery, 5 rats from each group at each time point were euthanized by intraperitoneal injection of an overdose of sodium pentobarbital (150 mg/kg). Maxillary bone blocks containing the first molars were immediately harvested and fixed in 4% paraformaldehyde solution at 4 °C for 48 h. After fixation, samples were scanned using micro-computed tomography (Micro-CT; Quantum GX2, PerkinElmer, United States) with a tube voltage of 90 kV and current of 88 µA. Data were exported in DICOM format and reconstructed in 3D Slicer software (version 5.9.0; RRID:SCR_005619). A standardized region of interest (ROI) was delineated at the site of pulp exposure to quantify the volume of newly formed reparative dentin bridges (Dentin Volume, DV) and the total pulp chamber tissue volume (Tissue Volume, TV). The DV/TV ratio was calculated to evaluate the rate of new dentin formation.

#### 2.9.2 Histological preparation and staining

Following Micro-CT scanning, the same specimens were decalcified in 10% EDTA solution at room temperature (22 °C ± 2 °C) for approximately 2 weeks, with fresh decalcifying solution renewed weekly. After complete decalcification, samples were dehydrated in graded ethanol, cleared in xylene, and embedded in paraffin. Serial sections of 5 µm thickness were cut along the mesio-distal axis using a rotary microtome. Sections were stained with hematoxylin and eosin (HE), and representative sections were additionally processed with Masson’s trichrome staining.

#### 2.9.3 Histological evaluation

All histological sections were digitized using a Leica Aperio digital slide scanner (Leica Biosystems, Nussloch, Germany). Histological evaluation was independently performed by two calibrated examiners who were blinded to group allocation throughout the assessment process to minimize bias.

Semi-quantitative histomorphometric scoring was carried out according to the histological criteria described by (Long et al., 2017). Parameters assessed included: (1) degree of inflammatory response in the pulp tissue; (2) continuity and thickness of the reparative dentin bridge; and (3) morphology and arrangement of the odontoblast-like cell layer beneath the dentin bridge. The complete scoring system is provided in Supplementary Table S2.

### 2.10 Statistical analysis

All quantitative data were expressed as mean ± standard deviation (mean ± SD). Statistical analyses were performed using GraphPad Prism 9.0 software (GraphPad Software, San Diego, United States,RRID:SCR_002798). Comparisons among multiple groups were conducted using one-way analysis of variance (ANOVA) followed by Tukey’s *post hoc* multiple comparison test. A value of P < 0.05 was considered statistically significant.

Histological results were treated as ordinal data. The Kruskal–Wallis H test was used to evaluate differences among groups. When statistically significant differences were detected, pairwise comparisons were further analyzed using the Mann–Whitney U test. A value of P < 0.05 was considered statistically significant.

## 3 Results

### 3.1 Physicochemical characterization of Sr-HA

**FIGURE 1:**
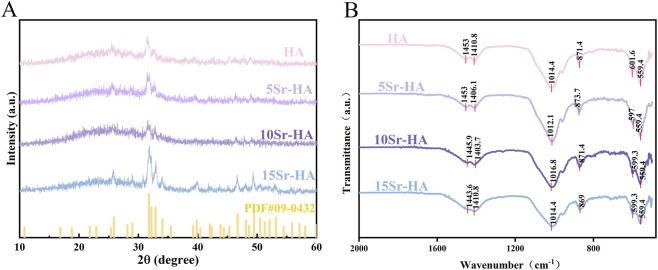
**(A)** XRD patterns of the corresponding samples, with the standard pattern for HA JCPDS No. 09-0432 shown at the bottom for reference. **(B)** FTIR spectra of pure HA and Sr-HA samples with different Sr substitution ratios (5%, 10%, and 15%).

Hydroxyapatite hollow microspheres with varying strontium (Sr) doping ratios were successfully synthesized via a one-step hydrothermal method. XRD patterns (Figure 1A) of pure HA and Sr-doped Hydroxyapatite (5Sr-HA,10Sr-HA,15Sr-HA) displayed characteristic peaks matching the standard HA card (JCPDS No. 09-0432). The dominant reflections at 2θ ≈ 25.9°, 31.8°, 32.2°, 32.9°, and 49.5° corresponded to the (002), (211), (112), (300), and (213) planes, respectively. No secondary phases were detected, confirming the phase purity of the synthesized samples. No secondary phases were detected in any group. A gradual shift of major reflections toward lower angles was observed as Sr content increased (Ren et al., 2022; Codrea et al., 2024).

FTIR spectra (Figure 1B) showed characteristic phosphate absorptions (ν_3_: ∼1,011–1,016 cm^−1^; ν_4_: ∼557–570 cm^−1^; ∼601 cm^−1^) and carbonate-related peaks at 1,406–1,452 cm^−1^. Minor variations in phosphate vibration bands were detected among the Sr-doped groups (Baldassarre et al., 2023).

**FIGURE 2:**
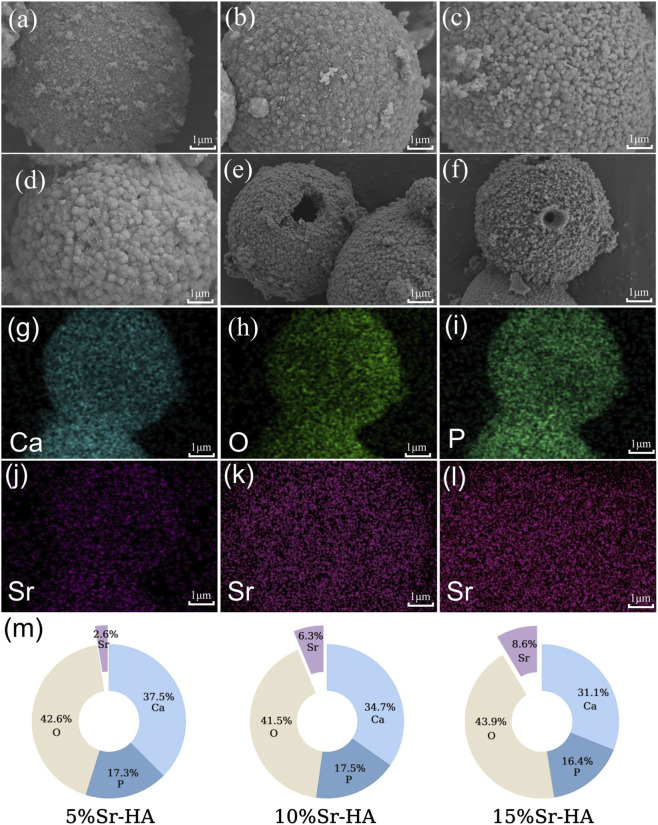
**(a–d)** SEM images of HA, 5%Sr-HA, 10%Sr-HA, and 15%Sr-HA showing uniform spherical morphology. **(e,f)** Fractured 5%Sr-HA microspheres revealing a hollow internal structure. **(g–j)** EDS elemental mapping of 5%Sr-HA confirming uniform distribution of Ca, O, P, and Sr. **(k,l)** EDS Sr mapping of 10%Sr-HA and 15%Sr-HA. **(m)** Quantitative EDS analysis.

SEM analysis revealed monodisperse microspheres with uniform spherical morphology and an average diameter of approximately 12–14 μm (Figures 2a–d). Fractured microspheres displayed a well-defined hollow core structure (Figures 2e,f). All Sr-doped microspheres maintained structural integrity without visible agglomeration.

EDS spectra confirmed the presence of Ca, P, O, and Sr in the doped samples (Figures 2g–j). Elemental mapping (Figures 2k,l) demonstrated homogeneous Sr distribution throughout the microspheres, indicating uniform dopant incorporation. Quantitative EDS analysis (Figure 2m) showed a progressive increase in Sr atomic percentage—from 2.6% (5% Sr-HA) to 6.3% (10% Sr-HA) and 8.6% (15% Sr-HA)—accompanied by a corresponding decrease in Ca content. This trend is consistent with partial substitution of Ca^2+^ by Sr^2+^ within the hydroxyapatite lattice.

### 3.2 Characterization and identification of hDPSCs

Before conducting subsequent functional experiments, we first verified the stem cell identity and purity of the isolated hDPSCs to ensure the reliability of the biological assessments. To confirm their mesenchymal stem cell characteristics, we evaluated cell morphology, immunophenotype, and multipotent differentiation capacity.

**FIGURE 3:**
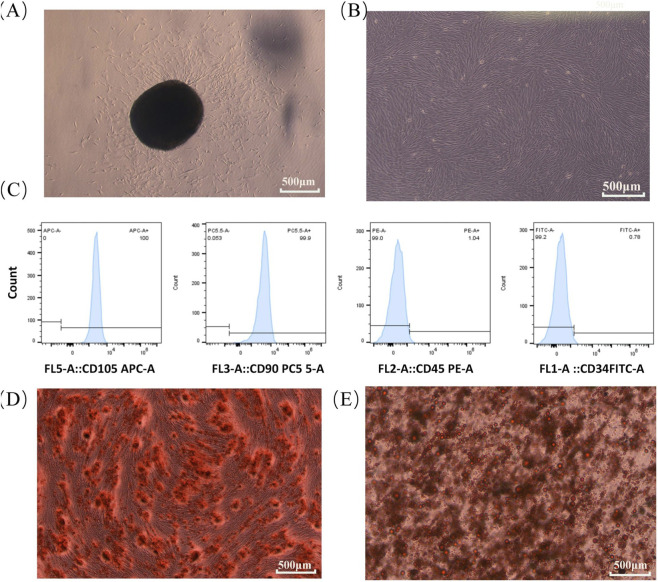
hDPSCs identification: **(A,B)** typical morphology; **(C)** flow cytometry confirming CD105^+^/CD90^+^ and CD45^−^/CD34^−^ phenotype; **(D,E)** osteogenic (ARS) and adipogenic (Oil Red O) differentiation.

Under an inverted microscope, primary hDPSCs exhibited adherent growth with a typical spindle-shaped, fibroblast-like morphology and demonstrated robust proliferative activity. Cells were expanded to passage 3 for subsequent assays (Figures 3A,B).

Immunophenotypic profiling by flow cytometry showed high expression of mesenchymal stem cell markers CD90 and CD105 (>95%), whereas hematopoietic markers CD34 and CD45 were nearly absent (<2%) (Figure 3C). This marker profile aligns with the minimal defining criteria for mesenchymal stem cells proposed by the International Society for Cell & Gene Therapy (ISCT) (Viswanathan et al., 2019).

Multipotent differentiation assays further confirmed the stem cell identity of the isolated cells (Figures 3D,E). After 21 days of osteogenic induction, Alizarin Red S staining revealed abundant mineralized nodules. Following 14 days of adipogenic induction, Oil Red O staining demonstrated numerous intracellular lipid droplets, indicating adipogenic differentiation potential.

Collectively, the morphological features, immunophenotypic profile, and confirmed osteogenic and adipogenic differentiation abilities demonstrate that the isolated cells were high-purity hDPSCs suitable for subsequent experiments.

### 3.3 Ion release profiles and screening of the optimal Sr doping concentration

**FIGURE 4:**
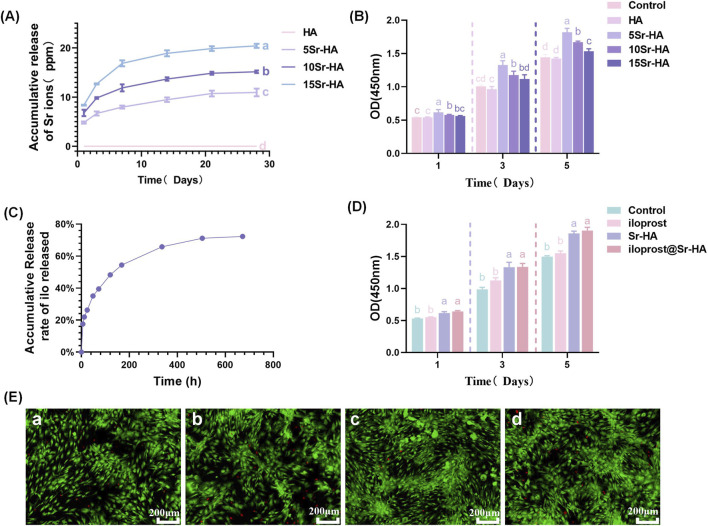
**(A)** Cumulative Sr^2+^ release from Sr-HA with different substitution ratios (independent samples at each time point, n = 3). **(B)** CCK-8 assay of Sr-HA groups, showing optimal proliferation at 5% Sr-HA. **(C)** Cumulative release profile of iloprost from Ilo@Sr-HA (independent samples at each time point, n = 3). **(D)** Proliferation comparison among groups, with Ilo@Sr-HA exhibiting the strongest effect. **(E)** Live/Dead staining: (a) Control; (b) Iloprost; (c) Sr-HA; (d) Ilo@Sr-HA. Green = live cells, Red = dead cells. Scale bar = 200 μm. Data in **(A–D)** are presented as mean ± SD (n = 3). For time-dependent experiments, statistical comparisons were performed separately at each time point using one-way ANOVA followed by Tukey’s *post hoc* test. Different lowercase letters indicate statistically significant differences among groups (P < 0.05). Groups sharing at least one common letter are not significantly different.

The cumulative Sr^2+^ release from Sr-HA with 0%, 5%, 10%, and 15% doping showed a biphasic pattern consisting of an initial burst within the first several days followed by a gradual, sustained release phase (Figure 4A). By day 21, the cumulative Sr^2+^ concentrations reached approximately 10.7 ppm, 14.9 ppm, and 19.9 ppm for the 5%, 10%, and 15% Sr-HA groups, respectively, and stabilized at approximately 10.9 ppm, 15.2 ppm, and 20.4 ppm by day 28. The undoped HA group showed negligible Sr^2+^ release throughout the testing period.

Cell proliferation assessed by the CCK-8 assay demonstrated a concentration-dependent response to Sr doping (Figure 4B). At days 1, 3, and 5, all Sr-HA groups exhibited higher OD values than the undoped control. Among the Sr-doped samples, the 5% Sr-HA group consistently showed the highest viability at days 3 and 5 (P < 0.05), whereas the 10% and 15% groups displayed lower proliferation than the 5% group and showed slight reductions at some time points.

Based on these observations, the 5% Sr-HA formulation was selected for subsequent experiments.

### 3.4 Loading and release of iloprost

The cumulative release of iloprost from 5% Sr-HA microspheres was quantified over a 720-h period (Figure 4C). Ilo@Sr-HA exhibited a biphasic release pattern, characterized by a rapid initial increase during the first 72 h followed by a gradual, sustained release phase. The cumulative release reached approximately 70%–75% at around 600 h, after which the release curve approached a plateau with minimal further change.

### 3.5 *In vitro* biocompatibility of microspheres

The cytocompatibility and metabolic activity of hDPSCs cultured with different materials were assessed using the CCK-8 assay (Figure 4D). At day 1, OD values were similar among all groups. By days 3 and 5, both the Sr-HA and Ilo@Sr-HA groups showed higher OD values compared with the control and iloprost-only groups. The Ilo@Sr-HA group exhibited the highest OD value at day 5, while the iloprost-only group demonstrated slightly elevated values relative to the control but remained lower than the Sr-containing groups.

Live/Dead fluorescence staining (Figure 4E) showed predominantly green fluorescence with minimal red fluorescence across all groups, indicating high cell viability and the absence of cytotoxic effects. Consistent with the CCK-8 results, the Ilo@Sr-HA group displayed a visually higher density of viable cells and more uniform cell spreading, whereas the control, iloprost-only, and Sr-HA groups showed comparatively lower coverage.

### 3.6 Effects of Ilo@Sr-HA on the odontogenic differentiation of hDPSCs

**FIGURE 5:**
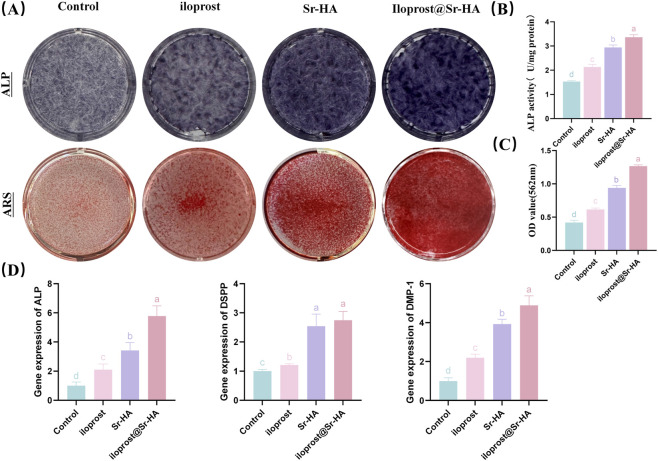
**(A)** ALP staining (Day 7) and ARS staining (Day 28). **(B)** Quantitative ALP activity on Day 7. **(C)** Quantification of mineral deposition by ARS on Day 28. **(D)** Gene expression of ALP, DSPP, and DMP1 on Day 5. Data in **(B–D)** are presented as mean ± SD (n = 3). Different lowercase letters above bars indicate statistically significant differences among groups (P < 0.05).

The osteo/odontogenic differentiation of hDPSCs in response to the different materials was evaluated by ALP activity, extracellular matrix mineralization, and the expression of odontogenic genes at defined time points (Figure 5). At day 7, ALP staining revealed notably stronger coloration in the 5% Sr-HA and Ilo@Sr-HA groups compared with the pure HA and control groups, with the most intense staining observed in the Ilo@Sr-HA group. Quantitative ALP measurement further confirmed that ALP activity in the Ilo@Sr-HA group was significantly higher than in all other groups (P < 0.05), indicating a more pronounced early-stage differentiation response at this time point.

Mineralized nodule formation was assessed after 28 days of induction. Alizarin Red S staining demonstrated that all groups formed calcified nodules, but the Ilo@Sr-HA group showed the greatest number and largest area of mineralized deposits. Quantitative analysis of the extracted ARS dye verified that the Ilo@Sr-HA group exhibited the highest degree of extracellular matrix mineralization at this late stage, significantly exceeding the Sr-HA, HA, and control groups (P < 0.05).

Odontogenic gene expression was examined at day 5 to capture early transcriptional changes preceding enzymatic and mineralization outcomes. qRT-PCR analysis revealed significantly upregulated expression of ALP, DMP1, and DSPP in the Ilo@Sr-HA group compared with the other groups (P < 0.05). Among these markers, DMP1 and DSPP—which are closely associated with dentin matrix formation—displayed the most pronounced increases in the Ilo@Sr-HA group, whereas the Sr-HA and HA groups showed comparatively lower expression levels.

### 3.7 Reparative dentin formation effects of Ilo@Sr-HA *in vivo*

**FIGURE 6:**
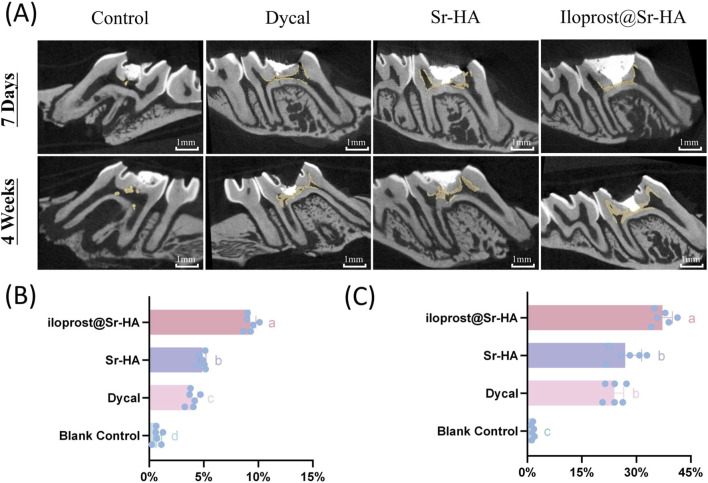
**(a)** Representative micro-CT sagittal sections of pulp-exposed molars in each group at 7 days and 4 weeks. Newly formed mineralized tissue is highlighted in yellow. Scale bar = 1 mm. **(B)** Quantification of dentin volume fraction (DV/TV) at 7 days. **(C)** DV/TV ratios at 4 weeks. Data in **(B,C)** are presented as mean ± SD (n = 10). Different lowercase letters indicate statistically significant differences among groups (P < 0.05)

Reparative dentin formation was evaluated using micro-CT, histological staining, and semi-quantitative scoring at early and late healing stages. Micro-CT analysis showed time-dependent increases in mineralized tissue across all treated groups (Figure 6). At day 7, the Ilo@Sr-HA group exhibited the highest dentin volume fraction (DV/TV), reaching 8.95%, which exceeded the values of the Dycal group (6.20%) and the Sr-HA group (5.38%), whereas the blank control displayed only minimal mineralized tissue. By week 4, DV/TV values increased in all treatment groups, with the Ilo@Sr-HA group reaching 38.91%, significantly higher than the Dycal (26.53%) and Sr-HA (23.23%) groups (P < 0.01).

**FIGURE 7:**
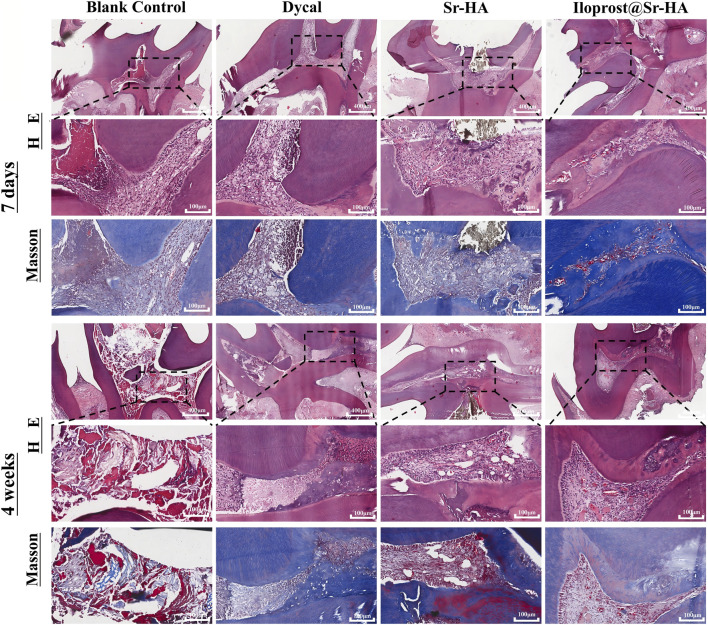
H&E and Masson staining of pulp-dentin response at 7 days and 4 weeks. Ilo@Sr-HA shows best inflammatory control and dentin bridge formation. Magnification: 5× (overview), 20× (detail).

Histological observations were consistent with these findings (Figure 7). At day 7, HE staining revealed mild inflammatory cell infiltration beneath the capping interface in both the Sr-HA and Ilo@Sr-HA groups, with the Ilo@Sr-HA group showing relatively preserved pulp architecture and fewer inflammatory cells. The Dycal group demonstrated moderate inflammation, whereas the blank control exhibited severe inflammatory infiltration and pulp necrosis. By week 4, the Ilo@Sr-HA group had formed a continuous and relatively thick layer of reparative dentin lined with odontoblast-like cells, while the Sr-HA group also showed bridge formation but with reduced thickness and continuity. The Dycal group displayed irregular or incomplete dentin bridge structures, and no bridge formation was observed in the blank control, which showed persistent necrosis.

Masson’s trichrome staining at week 4 further demonstrated that the newly deposited hard tissue in the Ilo@Sr-HA group contained densely packed collagen fibers with more mature staining features compared with the Sr-HA and Dycal groups. Neovascular-like structures were occasionally observed in both the Sr-HA and Ilo@Sr-HA groups; however, because no quantitative vascular assessment was performed, these findings are reported only as qualitative histological observations.

Semi-quantitative scoring results aligned with the micro-CT and staining data (Table 1) At day 7, the Ilo@Sr-HA group recorded the lowest inflammation scores and exhibited better pulp preservation than the Dycal and control groups (P < 0.05). Early-stage hard tissue formation scores did not differ significantly among the treatment groups, despite the micro-CT analysis showing higher DV/TV values in the Ilo@Sr-HA group at this time point. By week 4, the Ilo@Sr-HA group consistently achieved higher scores for dentin bridge continuity, thickness, and quality than the Sr-HA and Dycal groups (P < 0.05).

**TABLE 1:** Data are presented as median (interquartile range).

Time	Group	No. of specimen	Inflammatory cell response score	Hard tissue formation score	Quality of dentin formation score
7 days	Blank control	10	4.0 (4.0–4.0)^c^	4.0 (4.0–4.0)^b^	-
	Dycal	10	3.0 (2.0–3.0)^b^	2.5 (2.0–3.0)^a^	-
	Sr-HA	10	2.0 (2.0–3.0)^ab^	2.5 (2.0–3.0)^a^	-
	Iloprost@Sr-HA	10	1.0 (1.0–2.0)^a^	2.0 (2.0–2.0)^a^	-
4 weeks	Blank control	8	4.0 (4.0–4.0)^c^	4.0 (3.0–4.0)^c^	3.0 (3.0–3.0)^c^
	Dycal	10	2.0 (2.0–2.0)^b^	2.0 (2.0–2.0)^b^	2.0 (2.0–2.0)^b^
	Sr-HA	9	2.0 (2.0–2.0)^ab^	2.0 (2.0–2.0)^b^	2.0 (2.0–1.5)^ab^
	Iloprost@Sr-HA	10	1.0 (1.0–1.0)^a^	1.0 (1.0–1.0)^a^	1.0 (1.0–2.0)^a^

Different lowercase letters indicate statistically significant differences among groups. Groups sharing at least one common letter are not significantly different. (P < 0.05, Kruskal–Wallis test followed by Mann–Whitney U test).

## 4 Discussion

This study evaluated the biological performance of iloprost-loaded, strontium-doped hydroxyapatite microspheres and demonstrated that the combined material created a microenvironment conducive to early cellular activity and subsequent tissue repair within the pulp–dentin complex. The physicochemical characterizations provide important context for interpreting the biological performance of the materials. XRD analysis showed a gradual leftward shift of characteristic hydroxyapatite reflections with increasing Sr content, consistent with lattice expansion caused by the substitution of Ca^2+^ (0.99 Å) with the larger Sr^2+^ ion (1.18 Å) (Ren et al., 2022; Codrea et al., 2024). This shift, in the absence of secondary phase formation, suggests successful incorporation of Sr into the HA crystal lattice rather than surface adsorption. FTIR spectra further indicated subtle changes in phosphate-related vibrational bands, which may reflect localized structural distortions associated with Sr substitution (Baldassarre et al., 2023). SEM imaging confirmed that the hollow microsphere morphology was preserved across all doping levels, and EDS mapping demonstrated uniform Sr distribution, supporting the conclusion that Sr incorporation did not disrupt microsphere formation.

From a biological standpoint, the Sr^2+^ release profiles showed the expected concentration-dependent increase associated with higher doping levels, accompanied by a biphasic pattern typical of porous calcium phosphate systems (Alves Côrtes et al., 2024). Importantly, the 5% Sr-HA group exhibited moderate and sustained Sr^2+^ release, whereas higher doping levels (10% and 15%) produced substantially greater ion concentrations that were associated with reduced cell proliferation *in vitro* (Ran et al., 2023). These findings highlight that while Sr incorporation into HA can enhance bioactivity, excessive doping may alter the ionic microenvironment beyond the optimal range for hDPSCs viability. This is consistent with reports that Sr concentrations within a moderate range enhance stem cell activity, while excessive Sr may induce ionic imbalance and impair viability (Liu et al., 2023a; Kong et al., 2024). Our release data showed that 5% Sr-HA maintained Sr^2+^ levels around 10–11 ppm, whereas 10% and 15% Sr-HA produced higher concentrations accompanied by reduced proliferation, suggesting a dose-dependent effect. Therefore, both structural and biological observations support the selection of 5% Sr-HA as an optimal formulation for subsequent iloprost loading and functional evaluation.

*In vitro*, Ilo@Sr-HA enhanced multiple indicators of odontogenic differentiation, including alkaline phosphatase activity, extracellular matrix mineralization, and the expression of dentin-related genes such as ALP, DMP1, and DSPP. These findings suggest that the material provides favorable cues for both the initiation and progression of odontogenic differentiation. Although the underlying mechanisms were not investigated in detail, previous reports indicate that Sr-containing biomaterials can influence odontogenic gene expression (Huang et al., 2016; Mandakhbayar et al., 2019; Abdalla et al., 2022), while iloprost has been associated with improved cellular stability under stress conditions (Giordo et al., 2021). The enhanced differentiation observed here may therefore reflect the respective and potentially parallel contributions of ionic and pharmacologic cues, although the specific pathways involved remain to be clarified. The possibility of synergistic interactions is supported by recent evidence showing that strontium-doped hydroxyapatite can potentiate the biological activity of co-delivered therapeutic molecules, resulting in enhanced osteogenic and angiogenic responses (Liu et al., 2025).

The *in vivo* results further supported the reparative potential of Ilo@Sr-HA. Micro-CT analysis showed greater early mineral deposition at day 7 and a higher volume of reparative hard tissue by week 4 compared with Dycal and Sr-HA. Histological examination revealed reduced inflammation during early stages and the formation of a relatively continuous reparative dentin bridge at later stages. The newly deposited tissue in the Ilo@Sr-HA group displayed a more organized structure with aligned odontoblast-like cells, whereas Dycal tended to produce thinner or structurally irregular hard tissue (Arandi, 2017). Masson’s trichrome staining indicated denser and more mature collagen organization in the Ilo@Sr-HA group. Neovascular-like structures were occasionally observed; however, these findings were descriptive in nature as no angiogenesis-specific assessments were performed. Recent studies have shown that vascular endothelial cells can significantly enhance odontogenic differentiation of DPSCs through paracrine interactions, highlighting the importance of a pro-angiogenic microenvironment in dentin–pulp regeneration (Kong et al., 2024). Semi-quantitative histological scoring aligned with the imaging results and was consistent with improved inflammation control, tissue preservation, and dentin bridge morphology in the Ilo@Sr-HA group.

Taken together, the combined *in vitro* and *in vivo* findings collectively indicate that Ilo@Sr-HA supports early cellular activation and subsequent reparative dentin formation more effectively than Sr-HA alone. The sustained release of Sr^2+^ and iloprost may contribute to this favorable environment by providing prolonged exposure to biologically active cues. Nonetheless, further mechanistic investigations are necessary to clarify the specific pathways involved and to determine how these two components influence pulp tissue responses over time.

Several methodological and interpretative limitations should be taken into account when evaluating these findings. First, although neovascular-like structures were observed histologically, we did not perform angiogenesis-specific analyses, such as CD31/VEGF immunostaining or quantitative vessel assessment (Di et al., 2024). Therefore, these vascular observations should be considered qualitative rather than definitive evidence of angiogenic activity. Second, the *in vivo* observation period was limited to 4 weeks, which is adequate for assessing early reparative responses but not long-term dentin bridge maturation or pulp vitality. Extended follow-up studies will be required to determine the durability of the reparative outcomes. Finally, while the rat molar pulp-capping model is widely used for preliminary research, anatomical and physiological differences from human teeth may affect translational relevance (Aubeux et al., 2021). Further validation in larger animal models will thus be essential before clinical translation can be considered.

## 5 Conclusion

This study demonstrated that iloprost-loaded, strontium-doped hydroxyapatite microspheres provide a favorable environment for pulp–dentin complex repair. The material combined the structural advantages of hollow hydroxyapatite with the controlled release of Sr^2+^ and iloprost, supporting odontogenic differentiation *in vitro* and facilitating reparative dentin formation *in vivo*. Among the tested formulations, 5% Sr-HA offered the most balanced ionic release profile and cytocompatibility, forming the basis for the drug-loaded system. While these findings strongly highlight the potential of Ilo@Sr-HA as a biologically active pulp-capping material, further long-term and mechanistic studies—particularly those addressing angiogenesis and molecular signaling—are needed before translation to clinical scenarios can be considered.

## Data Availability

The datasets presented in this article are not readily available because no. Requests to access the datasets should be directed to 424053164@qq.com.
