# Supplementary material for: Strontium-doped hydroxyapatite microspheres loaded with iloprost promote dentin–pulp complex regeneration
Source: Front Bioeng Biotechnol. 2026 Jan 14;13:1726285. doi: 10.3389/fbioe.2025.1726285 (PMC12847251; doi:10.3389/fbioe.2025.1726285)
Supplement: Supplementary file 1 [file Supplementaryfile1.docx]

| GENE | Primer Sequence (5’–3’) |
| --- | --- |
| DMP1 | Forward: CACTCAAGATTCAGGTGGCAG  Reverse: TCTGAGATGCGAGACTTCCTAAA |
| DSPP | Forward: TGGCGATGCAGGTCACAAT  Reverse: CCATTCCCACTAGGACTCCCA |
| ALP | Forward: AACATCAGGGACATTGACGTG  Reverse: GTATCTCGGTTTGAAGCTCTTCC |
| GAPDH | Forward:GGCAAATTCAACGGCACAGTCAAG  Reverse: TCGCTCCTGGAAGATGGTGATGG |

Supplementary Tables 1 Relevant primer information

| Grade | Hard tissue formation | Inflammatory cell response | Quality of dentin formation in the bridge |
| --- | --- | --- | --- |
| 1 | Heavy: complete and continuous dentin bridge | Absent or few inflammatory cells | Regular pattern of dentinal tubules |
| 2 | Moderate: incomplete and discontinuous dentin bridge | Mild inflammation near dentin bridge or exposure area | Irregular dentinal tubules |
| 3 | Slight, scattered, or fuzzy hard tissue deposition | Moderate inflammatory cells in coronal pulp | No tubules present |
| 4 | No hard tissue deposition | Severe inflammation throughout coronal pulp | — |

Supplementary Tables 2 Criteria used for the histologic analysis of pulp response following direct pulp capping.
